# The chemotactic properties of various topical brimonidine tartrate ophthalmic preparations

**DOI:** 10.1186/s40360-020-0401-z

**Published:** 2020-03-23

**Authors:** Ruiz Simonato Alonso, Helena Parente Solari, Eduardo de França Damasceno, Miguel Noel Nascentes Burnier, Marcelo Palis Ventura

**Affiliations:** 1 Department of Ophthalmology, Fluminense Federal University, Niteroi, Rio de Janeiro Brazil; 2 The Henry C. Witelson Ocular Pathology Laboratory, McGill University, Montreal, Canada

**Keywords:** Brimonidine tartrate, Adverse effect, Benzalkonium chloride, Ophthalmic solutions, Glaucoma

## Abstract

**Background:**

The study aimed to evaluate and compare the leukocyte chemotactic activities of various brimonidine tartrate (BT) eye drop formulations.

**Methods:**

A 96-well dot-blot platet using a Boyden-style well was used to study the chemotactic effects of BT ophthalmic preparations. A modification was made to create blind wells where the tested agents were placed. Leukocytes were isolated from the peripheral blood of healthy volunteers. As positive controls, we used diluted drugs, benzalkonium chloride solution (BAK), zymosan-activated serum, and formyl-methionine-leucine-phenylalanine peptides. The negative control in our study was a phosphate-buffered saline solution. For each experimental condition, we measured leukocyte migration through a Millipore membrane. The differences in the mean migration distance between groups were compared using the analysis of variance (ANOVA).

**Results:**

The measured migration distances (in μm ± SD) were 62.14 ± 3.71 for BT 0.2% with BAK (Alcon Laboratories Inc.); 63.61 ± 3.81 for BT 0.2% with BAK (Allergan Inc); 40.36 ± 3.17 for BT 0.15% without BAK; and 41.02 ± 2.17 for BAK alone. The negative controls showed no chemotactic activity, while the positive controls showed the highest neutrophil migration of all experimental conditions. The differences between BT 0.15% without BAK and the other commercial formulations were statistically significant.

**Conclusion:**

Commercial ophthalmic preparations of BT 0.2% with BAK 0.005% had higher chemotactic properties than the alternative of a lower concentration of BT and without the preservative BAK. Therefore, the latter should be considered for patients with glaucoma or ocular hypertension in order to minimize iatrogenic ocular inflammation.

## Background

The inflammatory process is characterized by leukocyte invasion triggered by specific chemotactic factors. Leukocytes are an important cell type that both potentiate and sustain inflammation by releasing enzymes and producing various metabolites and inflammatory mediators. These include prostaglandins, leukotrienes, and platelet-activating factors. Leukocyte locomotion and chemotactic dynamics have been the subject of numerous studies over the past few decades. Chemotactic leukocyte migration has been described as the speed or rate of motion of cells through a matrix that is induced by substances in the environment (chemokinesis), or alterations in the vectorial movement of cells towards a stimulatory agent or chemoattractant (chemotaxis) [1–4].

Glaucoma and ocular hypertension are related eye conditions that result from a sustained increase in eye pressure that often leads to irreversible damage of the optic nerve fibers. The natural course of the disease, if left untreated, is permanent vision loss in the affected eye. Several treatments are recommended for glaucoma and ocular hypertension, including the formulation of brimonidine tartrate (BT) 0.2% (2.0 mg/mL) eye drops; which also includes the non-active ingredients benzalkonium chloride (BAK) 0.005% (0.05 mg/mL), citric acid, polyvinyl alcohol, sodium chloride, sodium citrate, and purified water. Hydrochloric acid and/or sodium hydroxide are also added to achieve a physiological pH of 6.4. BAK is the most commonly used antimicrobial preservative in topical ophthalmic solutions. The BT 0.2% formulation is an alpha-2 selective adrenergic agonist that has been widely used for the treatment of glaucoma and ocular hypertension since 1996 [1, 2]. Currently, there are several generic brands of BT, including some BAK-free formulations. BT lowers intraocular pressure (IOP) by reducing aqueous humor production and enhancing uveoscleral drainage [2, 3]. When used twice daily, its efficacy is comparable to timolol 0.5%, another commonly used medication to decrease IOP. However, BT has less severe chronotropic effects and is therefore considered an optimal treatment option, either as a monotherapy, adjunctive therapy, or as a substitute [4–6]. Nevertheless, BT has adverse effects that are more common than other drugs, such as dry mouth, eyelid edema, and a burning sensation in the treated eye [7, 8]. Other toxic side effects, such as chronic dermatitis and granulomatous conjunctivitis, have also been described; and often require the medication to be discontinued. Nevertheless, when compared to beta blockers, BT has still fewer systemic side effects [4, 9–11].

In the attempt of reduce side-effects, another eye drop formulation introduce to the market with a lower concentration of BT at 0.15% (1.5 mg/mL) (Alphagan P©; Allergan Inc., Irvine, CA, USA) and contained stabilized oxychloride complex (SOC) 0.005% (0.05 mg/mL), instead of BAK as a preservative. This formulation also includes the inactive ingredients sodium carboxymethylcellulose, sodium borate, boric acid, sodium chloride, potassium chloride, calcium chloride, magnesium chloride, purified water, and hydrochloric acid and/or sodium hydroxide to adjust the pH to 7.4 [12, 13]. BAK at high concentrations can be toxic and can persist for long periods in ocular tissue, eventually leading to dose-dependent cell death [14–17]. BT 0.15% also has a 25% reduction of the active drug in the formula, and animal studies suggest that brimonidine has a higher bioavailability when mixed with SOC [18]. Several studies have demonstrated that BT 0.15% with SOC has the same efficacy as BT 0.2% with BAK for reducing IOP [19–21]. Moreover, a single study reported that the drug is more effective in dark brown irides [22].

Long-term use of topical glaucoma medications associated with preservatives can induce changes in the ocular surface and conjunctival cell infiltration [23–25]. Additionally, a common secondary complication of hypotensive eye drops is iatrogenic inflammation. Prior studies have shown that various topical glaucoma medications, such as prostaglandin analogues, beta blockers, cholinergic agonists, and carbonic anhydrase inhibitors used for the treatment of glaucoma, have leukocyte chemotactic effects [26]. Our experiment reproduces this technique to specifically investigate the inflammatory characteristics of BT. Leukocytes are central to the initiation and maintenance of inflammation through the release of enzymes and the production of inflammatory mediators. Therefore, IOP treatment with chemoattractant compounds may indirectly lead to inflammation via increased leukocyte migration [27].

Beta blockers, cholinergic agonists, and carbonic anhydrase inhibitors were once the mainstay of glaucoma therapy. However, over the last two decades, the pharmacological management of glaucoma and ocular hypertension has changed with the introduction of prostaglandin analogs. The International Council of Ophthalmology Guidelines for Glaucoma Eye Care now includes Latanoprost 50 μg/mL alongside timolol 0.25% or 0.5% as the two essential topical intraocular pressure-lowering medications [28]. Nevertheless, because monotherapy increases compliance, prostaglandins are currently recommended to initiate treatment as they achieve the highest reduction in IOP per a meta-analysis of randomized clinical trials [29]. Moreover, prostaglandin analogs are administered once per day, have accepted safety profiles and are currently recommended by the American Academy of Ophthalmology Preferred Practice Patterns [30]. In this new context, BT is used more broadly as a single therapy or in combination with drugs when compared to beta blockers and carbonic anhydrase inhibitors.

There are also several BT formulations that contain a variety of concentrations and preservatives that may have different chemotactic properties. To date, there has been little investigation into these newer glaucoma and ocular hypertension therapies, particularly with respect to their chemotactic properties that may lead to inflammation. Our study therefore examined the chemotactic properties of various BT formulations with regards to leukocyte migration to determine if any are better suited for avoiding undesirable inflammatory complications. This will lead to improved recommendations for the treatment of ocular hypertension and better outcomes for patients.

## Methods

### Leukocyte isolation

Per the rules of the national ethics research commission, in approved number project 1.466.576, blood was collected from 14 healthy donors, aged 21 to 55 years, in heparinized vacutainer tubes. An equal amount of 20 mg/mL Ficoll 400 (Pharmacia, Upsala, Sweden) was mixed with the collected blood, a deteriorated composite solution with a density of 1.077 g/mL. During the period of 30 to 45 min, the blood collected was exposed to room temperature to allow the red blood cells to sediment. The upper layer containing leukocytes was isolated and centrifuged at 400 g at room temperature for 10 min. The supernatant was removed and discarded, and then the leukocytes were washed twice with RPMI-1640 medium (Gibco BRL, Burlington, Canada) containing 5% foetal calf serum (FCS; ICN Pharmaceuticals Canada Ltd., Montreal, Canada). Finally, the concentration of leukocytes was adjusted to 2.5 × 10^6^ cells/ml in RPMI. Within 30 min after purification, the leukocytes obtained from collection were used to evaluate the characteristics of the migration of chemotactic properties.

### Drug formulations and controls

A 1:100 dilution was used to prepare the drugs for the experiment. We used zymosan-activated serum (ZAS) and the peptide formyl-methionine-leucine-phenylalanine (f-Met-Leu-Phe or fMLP) as positive controls. The negative control used was phosphate-buffered saline solution (PBS). Zymosan-activated serum was prepared by adding zymosan (Sigma-Aldrich, St. Louis, MO, USA) to fresh defibrinated human serum at a concentration of 1.35 mg/ml serum. In each of the tests, fresh frozen aliquots were used. f-Met-Leu-Phe (Sigma-Aldrich Chemical, St. Louis, MO) was used in concentrations ranging from 6 to 10 mg/ml to 100 mg/ml.

### Migration assays

To test the migration of leukocytes and chemotactic properties, a modified 96-well dot-blot plate (Bio-Rad Laboratories Inc., Hercules, CA, USA) was used as a Boyden-style blind well chemotaxis chamber. A modification was made to create blind wells where the tested agents were placed. A polyethylene barrier was sealed with firmly pressed solid silicone. Twenty-five microliters of each agent to be assayed was placed in each of the lower wells. We finely fit two 9 cm Millipore membranes (3  μmpore size) (Millipore Corporation, Bedford, MA, USA) to the rectangular collector and placed them in the lower collector containing the chemoattractive solution. A thorough visual inspection was carried out to ensure consistent suitability, without the possibility of wrinkles on the membrane surface, ensuring homogeneous conditions in the wells. Leukocyte suspensions (100 μL) containing an estimated 250.000 cells were placed in each of the upper wells. Finally, the entire assay of the chemotactic apparatus was incubated in a humidified atmosphere containing 5% CO_2_ for 90 min at a controlled temperature at 37 °C.

### Membrane staining technique

After incubation, a 0.85% NaCl solution was used to gently rinse the membrane, which was subsequently fixed for a period of 15 s in 100% ethanol and then rinsed with distilled water. Mayer’s haematoxylin was used as a rinsing solution for another 6 min, and the membrane was washed with water and bleached with acid alcohol for another 1 min. After washing with water one more time, the membrane was exposed to mild alkali for 2 min. The membranes were let to dry overnight at a controlled temperature of 37 °C. The next day, the membrane was made transparent after being subjected to an immersion bath in 100% xylene solution to finally be mounted on a glass slide for reading under a calibrated microscope.

### Measuring leukocyte migration

**Fig. 1:**
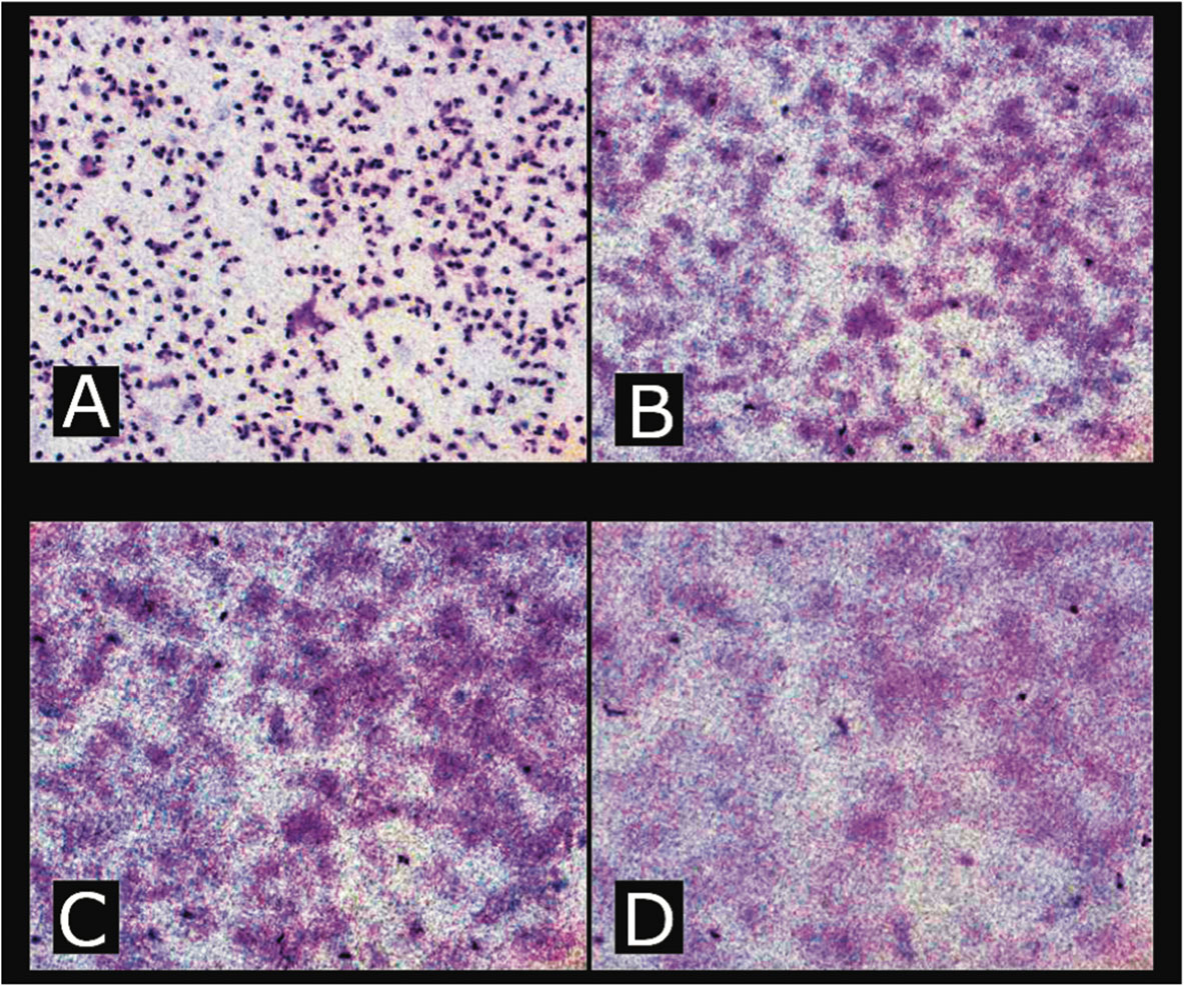
Leukocyte migration was evaluated using a Millipore membrane (Millipore). Migration was measured using a microscope where the Vernier graduated in micrometres (μm) was calibrated to obtain the measurements. The microscope was focused onto the cells on the upper surface of the membrane (**a**) and the position of the fine focus Vernier recorded. The depth of focus was then advanced through the membrane (**b** and **c**) until the last and furthest migrating cells were seen. At that position, the mark on the calibrated Vernier was again recorded (**d**). All photographs taken at 100× magnification

Leukocyte migration was evaluated using a Millipore membrane (Millipore). Migration was measured using a microscope where the Vernier graduated in micrometres (μm) was calibrated to obtain proper measurements at 100× magnification. Initially, we observed the cells on the upper surface, and the first mark was recorded. Then, we moved the focus into the membrane until the most distant leukocytes were identified, and this new position at the Vernier was recorded. The migration distance was established as the difference between the two registered positions. Five independent readings were taken for each sample considering different cells in the initial position. Each dilution was checked twice, and the average migration distance was used in our statistical analysis (Fig. 1).

### Statistical analysis

ANOVA (analysis of variance) was used to determine if the observed differences in mean migration distance were significant. A post hoc least significant difference (LSD) test was used for multiple comparisons. Values of *p* < 0.01 were considered statistically significant. The SPSS statistical software version 21.00 (IBM Corp., Armonk, NY, USA) was used for all statistical analyses.

## Results

The measured migration distances (μm ± SD) were 62.14 ± 3.71 for BT 0.2% with BAK (Alcon Laboratories Inc); 63.61 ± 3.81 for BT 0.2% with BAK (Allergan Inc); 40.36 ± 3.17 for BT 0.15% without BAK and 41.02 ± 2.17 for BAK alone. The negative controls showed no chemotactic activity, while the positive controls showed the highest neutrophil migration of all experimental conditions. The differences between BT 0.15% without BAK and the other commercial formulations were statistically significant (*p* < 0.01) (Tables 1 and 2).

**Table 1:** Effects of different brimonidine eye drops formulations on neutrophil migration

Drugs (1:100 dilution)	Migration distance ± SD (μm)
**BT 0.2% with BAK** (Alcon Inc)^a^	62.14 ± 3.71
**BT 0.2% with BAK** (Allergan Inc)^a^	63.61 ± 3.81
**BT 0.15% without BAK** (Allergan Inc)^b^	40.36 ± 3.17
**BAK-S**^b^	41.02 ± 2.17
**PBS**^c^	37.57 ± 2.14
**ZAS**^d^	77.21 ± 3.95
**fMLP**^d^	100.71 ± 3.94

(Mean migration ± standard deviation in **μm**)

^a^Drugs inducing significant chemotaxis (*p* < 0.01 versus PBS controls)

^b^Drugs inducing no significant chemotactic effect (*p* > 0.05 versus PBS controls)

^c^Negative control

^d^Positive controls

**Table 2:** Comparison among alpha 2-adrenergic agonists effects on neutrophil migration

Drugs	Mean difference	Standard error	Significance
BT without BAK versus BT with BAK (Allergan Inc)	- 23.25	1.26	*P* = 0.00*
BT without BAK versus BT with BAK (Alcon Inc)	- 21.78	1.26	*P* = 0.00*
BT with BAK (Allergan Inc) versus BT with BAK (Alcon Inc)	1.46	1.26	*P* = 0.25

^*^Statistically significant difference, post hoc Least Significant Difference (LSD), *p* < 0.01 was considered significant

**Fig. 2:**
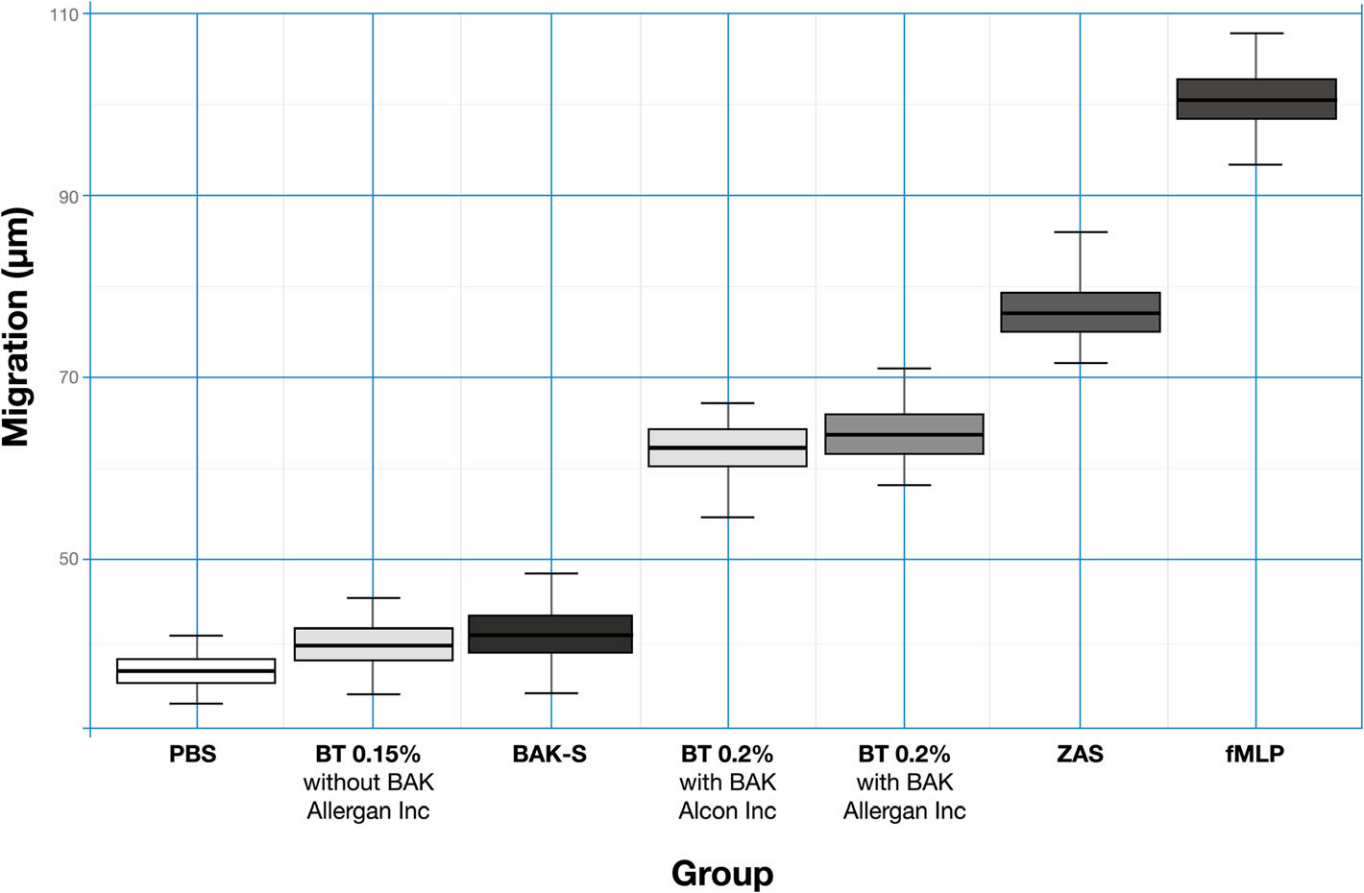
X-axis: Migration through the matrix measured in micrometers (μm); Y-axis: each experimental condition, including controls. BT 0.15% without BAK and BAK 0.005% alone had no significant chemotactic effect, while BT 0.2% with BAK 0.005% (Alcon Laboratories Inc.) and BT 0.2% with BAK 0.005% (Allergan Inc.) showed significantly higher chemotactic activity. Both the positive controls, ZAS and fMLP, showed the highest chemotactic effect, demonstrated by the longest migration distances of neutrophils through the matrix, as expected. Cells in the PBS negative control showed a negligible migration

BT 0.15% without BAK and BAK 0.005% solution had no significant chemotactic effects, and the difference between these two experimental conditions was not significantly different (*p* = 0.25). Both the positive controls, ZAS and fMLP, showed a significant chemotactic effect and had the longest migration distances. The PBS negative control showed a negligible migration distance (Fig. 2).

## Discussion

The study of the inflammatory response often requires the isolation of leukocytes, a key cell type responsible for the recognition and elimination of harmful substances. Leukocytes are intimately involved in the process of acute inflammation. Various methods have been described to isolate circulating leukocytes, including density centrifugation, flow cytometry, and spontaneous sedimentation. Several methods employ density gradients, especially for the isolation of neutrophils, blood cells, and polymorphic nuclear leukocytes.

The presence of chemotactic compounds, as preservatives and/or excipients is a significant factor associated with the inflammatory potential of many commercial ophthalmic preparations. Avoiding these drugs or adjusting treatment intervals may help to eliminate or decrease the consequent iatrogenic inflammation. This factor is especially important in patients who already have chronic ocular inflammation due to other conditions. An alternative approach to minimize chemotactic activity is to reformulate the concentration of active compounds in topical ophthalmic preparations, thereby reducing inflammation, enhancing compliance and ultimately higher rates of therapeutic success.

Three previous studies have investigated the role of topical pharmaceutical agents on leukocyte migration [26, 31, 32]. However, only one examined multiple antiglaucomatous drugs, including beta blockers, cholinergic agonists and nonselective adrenergic agonists [26]. To the best of our knowledge, our study is the first to examine the role of different alpha-2 selective adrenergic agonist (brimonidine) concentrations on neutrophil migration. Previously, BT has been associated with the development of anterior uveitis in patients, supporting our data that suggest that the drug has strong chemoattractant properties when compared to other formulations [33–35].

The cytotoxic effects of excipients used in ophthalmic formulations on human corneal epithelial cells have previously been related to prostaglandin analogs. In vivo and in vitro studies have shown that BAK induces both concentration-dependent and time-dependent cytotoxicity to the human corneal epithelium. Macrophage infiltration in the eyelids and visible defects in conjunctival cells were also described. Various ocular side effects of prostaglandin have been described in the literature, including foreign body sensation, ocular pruritus, a decrease in vision, reactivation of uveitis, herpes infection of the cornea, bacterial keratitis, and swelling of the retina (macular edema). Additionally, glaucoma patients treated with topical prostaglandin analogs have a higher incidence of dry eye syndrome and meibomian gland dysfunction. Yet, we could not find any published study on the leukocyte activity associated with prostaglandin analogs, one of the most frequently prescribed drugs for glaucoma treatment and a known proinflammatory molecule. We reiterate that the chemotactic properties of BAK, prostaglandins and other active and nonactive ingredients of hypotensive eye drops should continue to be the subject of future laboratory studies [36–38].

In our study, BT 0.15% with 0.005% SOC had no measurable chemotactic activity compared to the negative controls, such as BAK 0.005% solution. Our data suggests that BT formulations have a dose-dependent activation of the mediators of inflammation [13–15, 17, 23]. Therefore, the toxicity of a topical medication could be avoided by keeping the concentration of the active drug, BT in our case, under a certain threshold (Tables 1 and 2).

Several commonly used ophthalmic agents include BAK as a preservative and have been shown to increase neutrophil migration [26, 33]. Those include mydriatics, corticosteroids, nonsteroidal anti-inflammatory agents, antiallergy medications, antiglaucomatous agents, and some artificial tears. As these drugs are often used on a chronic basis, this inflammatory effect is clinically relevant, especially in patients with glaucoma or ocular surface disease (OSD). This has led to the development of less toxic preservatives to replace BAK, including detergents such as polyquaternium-1 (Polyquad®; Alcon Laboratories Inc.). In addition, newer classes of preservatives have been developed, such as stabilized oxidizing agents (stabilized oxychloro complexes [SOCs]) and ionic-buffered preservatives (SofZia®; Alcon Laboratories Inc.) [35, 39].

It is important of consider BAK when choosing the ideal eye drop for a patient with glaucoma, because those patients often use eyedrops that also contain BAK for different ocular conditions, such as Ocular surface disease (OSD). OSD is a common comorbidity in glaucoma patients, and its prevalence increases with age [40–42]. Artificial tears and antiglaucoma medications are frequently used for prolonged periods of time, sometimes even for an entire lifetime. Several authors have demonstrated that BAK can induce tear film instability, ocular surface damage, a decrease in the number of goblet cells, an increase in macrophage and fibroblast counts in the conjunctiva, and an increase in the expression of inflammatory markers on the conjunctiva [43]. These effects can lead to ocular discomfort, poor intraocular pressure control, glaucoma surgery failure, and decreased patient compliance [44].

Subclinical inflammation present in conjunctival and subconjunctival space caused by fibroblast activation and inflammatory cell infiltration has also been largely described in studies with patients receiving antiglaucoma therapy for long periods of time. Our findings support the idea that reduced-dose BT without BAK is a better option than BT with BAK for patients with glaucoma and ocular hypertension and could minimize the risk of inflammation. Choosing a formulation less prone to inflammation is also important when the drug is prescribed postoperatively, as some patients require complementary topical therapy after glaucoma surgery to achieve the target pressure and reduce symptoms. An increased inflammatory response during the healing phase and subsequent subconjunctival fibrosis may block aqueous outflow and lead to surgical failure [43, 45]. There is indeed an increasing amount of evidence from clinical and experimental studies that the long-term use of topical drugs can increase the potential risk of treatment failure after further glaucoma surgery due to chronic ocular surface changes.

## Conclusion

In summary, several studies have demonstrated that the use of ocular medications can cause significant iatrogenic effects on the eye. Therefore, when recommending an ocular hypotensive drug, the leukocyte chemotactic activities induced by antiglaucomatous formulations should also be considered, in order to reduce associated inflammation. In our study, we demonstrated that a formulation with a lower dose of BT without BAK showed no significant chemotactic effects, while the BT formulations with BAK showed statistically higher leukocyte chemotactic activity. Choosing the former should minimize iatrogenic inflammatory processes in patients with glaucoma or ocular hypertension, decreasing side-effects, increasing compliance and treatment success.

## Data Availability

The datasets used and/or analyzed during the current study available from the corresponding author on reasonable request.

## Abbreviations

BT: Brimonidine tartrate
BAK: Benzalkonium chloride
IOP: Intraocular pressure
SOC: Stabilized oxychloride complex
FCS: Fetal calf serum
BAK-S: Benzalkonium chloride solution
ZAS: Zymosan activated serum
fMLP: Formyl-methionine-leucine-phenylalanine peptides
PBS: Phosphate buffered saline
μm: Micrometers
LSD: Least significant difference
OSD: Ocular surface disease
